# Semantics bias in cross-national comparative analyses: is it good or bad to have “fair” health?

**DOI:** 10.1186/s12955-016-0469-8

**Published:** 2016-05-04

**Authors:** Christina W. Schnohr, Inese Gobina, Teresa Santos, Joanna Mazur, Mujgan Alikasifuglu, Raili Välimaa, Maria Corell, Curt Hagquist, Paola Dalmasso, Yeva Movseyan, Franco Cavallo, Saskia van Dorsselaer, Torbjørn Torsheim

**Affiliations:** Department of Public Health, University of Copenhagen, P. O. Box 2099, Copenhagen, Denmark; Department of Public Health and Epidemiology, Riga Stradins University, Riga, Latvia; William James Center for Research/Instituto Superior de Psicologia Aplicada-ISPA, Lisbon, Portugal; Institute of Mother and Child, Kasprzaka 17a str., Warsaw, Poland; Department of Pediatrics, Cerrahpasa Medical Faculty, Istanbul University, 34 303 , Fatih, Istanbul, Turkey; Department of Health Education, University of Jyväskylä, Jyväskylä, Finland; Folkhälsomyndigheten, 830140 Östersund, Sweden; Fakulteten för hälsa, natur- och teknikvetenskap, Karlstad University, 651 88 Karlstad, Sweden; Department of Public Health and Paediatrics School of Medicine, University of Torino, Via Santena, 10126 Turin, Italy; Arabkir Medical Centre-Institute of Child and Adolescent Health, Yerevan, 0014 Armenia; P.O. Box 725, 3500 AS Utrecht, The Netherlands; Institut for Samfunnspsykololgi, Kristie Bergiesgatan, Unversity of Bergen, Bergen, Norway

**Keywords:** Health Behavior in School-aged Children (HBSC), International comparison, Self-rated health, Measurement variance, Translation

## Abstract

The Health Behavior in School-aged Children is a cross-national study collecting data on social and health indicators on adolescents in 43 countries. The study provides comparable data on health behaviors and health outcomes through the use of a common protocol, which have been a back bone of the study sine its initiation in 1983. Recent years, researchers within the study have noticed a questionable comparability on the widely used item on self-rated health. One of the four response categories to the item “Would you say your health is….?” showed particular variation, as the response category “Fair” varied from 20 % in Latvia and Moldova to 3–4 % in Bulgaria and Macedonia. A qualitative mini-survey of the back-translations showed that the response category “Fair” had a negative slant in 25 countries, a positive slant in 10 countries and was considered neutral in 9 countries. This finding indicates that there are what may be called semantic issues affecting comparability in international studies, since the same original word (in an English original) is interpreted differently across countries and cultures. The paper test and discuss a few possible explanations to this, however, only leaving to future studies to hold a cautious approach to international comparisons if working with the self-rated health item with four response categories.

## Findings

- The item on self-rated health is a widely used indicator of health status as well as a predictor of health outcomes.
- International studies compare results from questionnaire surveys using the English version of self-rated health.
- The present study documents how the item responses vary across languages, which is not noticed in an English back translation.
- Hence, studies comparing self-rated health across different languages are likely to be affected by variation due to semantics bias.

## Introduction

The Health Behaviour in School-aged Children (HBSC) is a large cross-national survey consisting of questionnaire data on adolescents from 43 countries in Europe and North America. The survey was initiated in 1983, and for more than 30 years, the HBSC has provided researchers and policy makers with important knowledge on young people’s health and health behaviour. Validation of items in relation to comparability is an ongoing process for all studies within public health research, and in particular for international studies. Thus, international studies should continuously work for improving the validity of their questionnaire, and doing so by scrutinizing the translations of the items in use. Many studies within public health makes use of a general item on a person’s self perceived health, since it has shown to relate to various health behaviours, and has shown to be strongly associated to morbidity and mortality [1, 2]. The item is termed *self rated health*, and is often phrased *“Would you say your health is…?”* with four response categories of *“Excellent”*, *“Good”*, *“Fair”* and *“Poor”*.

This item has proven to work well in large epidemiological surveys [2]. In the process of validating this item prior to the HBSC-survey performed in 2005/06, the researchers found inconsistencies in the culturally specific interpretations of the category “fair”, leading to poorer comparability between the countries included in the HBSC as a whole.

The purpose of this study is to illustrate the inconsistencies identified, and make recommendations on future use of the item on self rated health in international studies.

## Methods

HBSC is conducted in collaboration with the World Health Organization Regional Office for Europe. The study collects data on social and health indicators as well as health behaviors. The study provides comparable data on young people’s health and lifestyle from countries with different societal and political systems through the use of a common protocol.

The HBSC study consists of repeated cross-sectional cluster sampled surveys among 11-, 13- and 15-year-old school children in representative samples of approximately 1 500 students from each of the three age groups in schools across the participating countries. The students fill in a standardized questionnaire during a school lesson after instruction from the teacher or researcher. HBSC has been collecting data on adolescents every fourth year since 1983. The HBSC study, on which the present paper is based, included 43 countries and regions with a total of 214.028 students in its latest survey in 2013/14. Further methodological issues related to the HBSC as a cross-national survey has been discussed elsewhere [3, 4].

Global self-reported health has been shown in many studies to be an independent predictor of mortality, even after accounting for known demographic, social and medical risk factors. Twenty-seven community studies have shown impressively consistent findings in relating self-reported health with future mortality, which persists when numerous health indicators and other relevant covariates are included in the analysis [2]. Some studies have also addressed the relationship between self-reported health and cause-specific mortality [1], indicating that a high number of causes are specifically associated with perceived health. In particular, diabetes, infectious and respiratory diseases show a strong association, while so-called social pathologies (accidents, suicides, and homicides) were not. The relationship with gender has also been explored, pointing out a quite strong gender difference which should be explored in more depth. It seems therefore quite important to maintain focus on self-reported health, given the fact that gender differences in perceived health have already been documented in the HBSC data and this domain of research seems to be a most interesting one for the future.

## Results

**Fig. 1:**
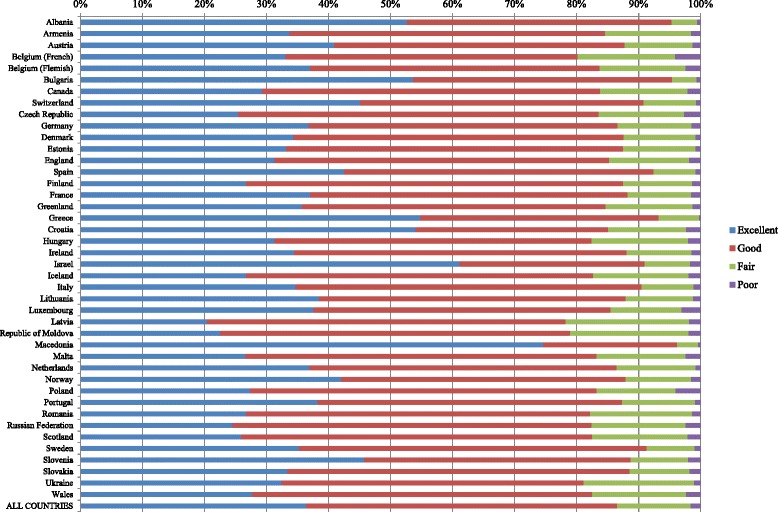
Response categories to the item on self-rated health across HBSC countries

Out of the 214.028 adolescents who had responded to the question *“Would you say your health is…?”* in 2013/14, a total of 77.917 (36.4 %) responded *Excellent*, 107.364 (50.2 %) responded *Good*, 25.269 (11.8 %) responded *Fair* and 3.478 (1.6 %) responded *Poor* (data not shown). However, there was a large variation across countries in the distribution of the four responses across the 42 HBSC countries as shown in Fig. 1.

As the colours indicate, there are large variations across the four categories. The response category *Fair* varies from 19.9 % in Latvia and 19.1 % in the Republic of Moldova, to 3.9 % in Bulgaria and 3.1 % in Macedonia.

A qualitative survey of the back-translations was performed among the responsible Principal Investigators (PI’s) were asked to report what semantic attribution they would apply to their national translation; whether it had a positive slant, a negative slant or was neutral. Out of the 44 replies obtained, the response category “fair” had a negative slant in 25 countries, a positive slant in 10 countries, and was considered neutral in 9 countries.

To test if there was a systematic variation due to socio-economic or cultural background, cross-tabulation with Chi^2^-test was run on slant versus Hofstede country classification by the cultural dimension (as used in the GLOBE study), and slant versus economy (in two categories). None of the tests showed significant association (*p*-value = 0.270 on country classification and p-value = 0.362 on economy).

## Discussion

This study found large variation in the response categories to the item on self-reported health with four response categories. The observed variation potentially bias interpretation, if study findings would compare response categories directly. The focus on the response with the potential to both a negative and a positive slant, showed variation from 3–19 %, which is large enough variation to indicate that semantics plays a role in the responses chosen.

The responses may be attributed to societal or cultural differences, which seems plausible when considering the difference between being in an English speaking country responding neutrally to the often posed question “how are you?” versus Northern European countries where you downplay your complaints and Eastern European countries, where it is common to express your complaints (do note that these thoughts are highly subjective and the view of the authors personally).

Given the seemingly large differences, the countries were classified into cultural clusters as used in the GLOBE study [5], which divided the included countries in ten clusters. In the present analyses, the countries were divided into the following clusters (with the number of countries in parenthesis; Anglo (4), Eastern Europe (7), German (5), Latin-Europe (7), Middle East (1) and Nordic (8). A Chi^2^-test of the association between cultural cluster and slant showed a p-value of .362. A second association was tested between the 24 countries with a high or middle-high economy and the 20 countries with a middle low economy, providing a p-value of .270. However simple statistical testing, we had to reject a societal or cultural dimension as a bias in responses to the “fair”-category in the self-rated health item for now.

There seems to be no doubt, that personal interpretations play a role in the matter; Most countries based their reply from a discussion in their research team, and several PI’s reported that their response was based on a consensus obtained even though there had been different opinions about the “most correct” answer.

An international study like HBSC has a general methodological challenge, and a raised public profile in scientific as well as policy areas calls for increased attention on improving the general data quality. Since survey quality is closely related to survey measurements [6], methodological issues and efforts to achieve comparability is an overall goal in the HBSC network. A predominant challenge when doing international surveys is to have member countries recognize the importance of mutual methodological issues [7], and future collaboration in the HBSC is aiming to take this on as a continuing challenge. In the future, this will be done by adapting to knowledge gain from the translation/back-translation procedures, but also consistently validating items qualitatively and including adolescents and for future studies also experts of linguistic and semantics.

This study pointed to the challenge in handling issues beyond translation when evaluating comparability in international studies. Secondly, one should hold a cautious approach when comparing the item self-reported health internationally. The authors propose only two solutions to meet the methodological challenge posed in the present paper partly overcoming the weaknesses if the category “fair” stands alone.

1. Either that the category “fair” is either combined with both “good” and “excellent” if the particular interest is the population reporting “poor” health, or
2. That the category “fair” is combined with “poor” and “good”, if the particular interest is the population reporting “excellent” health.

As a concluding remark, we wish to reply to the question posted in the title of the paper; whether it is good or bad to have “fair” health depends on which language you speak.

## Abbreviation

HBSC: Health Behavior in School-aged Children
